# Applying Nanoparticle Tracking Analysis to Characterize the Polydispersity of Aggregates Resulting from Tannin–Polysaccharide Interactions in Wine-Like Media

**DOI:** 10.3390/molecules24112100

**Published:** 2019-06-03

**Authors:** Sijing Li, Kerry L. Wilkinson, Agnieszka Mierczynska-Vasilev, Keren A. Bindon

**Affiliations:** 1 School of Agriculture, Food and Wine, The University of Adelaide, PMB 1, Glen Osmond, SA 5064, Australia; sli@csu.edu.au (S.L.); kerry.wilkinson@adelaide.edu.au (K.L.W.); 2 The Australian Research Council Training Centre for Innovative Wine Production, PMB 1, Glen Osmond, SA 5064, Australia; 3 The Australian Wine Research Institute, PO Box 197, Glen Osmond, SA 5064, Australia; Agnieszka.Mierczynska-Vasilev@awri.com.au

**Keywords:** mannoprotein, arabinogalactan, seed tannin, aggregation, nanoparticle tracking analysis

## Abstract

Interactions between grape seed tannin and either a mannoprotein or an arabinogalactan in model wine solutions of different ethanol concentrations were characterized with nanoparticle tracking analysis (NTA), UV-visible spectroscopy and dynamic light scattering (DLS). NTA results reflected a shift in particle size distribution due to aggregation. Furthermore, the light scattering intensity of each tracked particle measured by NTA demonstrated the presence of aggregates, even when a shift in particle size was not apparent. Mannoprotein and arabinogalactan behaved differently when combined with seed tannin. Mannoprotein formed large, highly light-scattering aggregates, while arabinogalactan exhibited only weak interactions with seed tannin. A 3% difference in alcohol concentration of the model solution (12 vs. 15% *v*/*v*) was sufficient to affect the interactions between mannoprotein and tannin when the tannin concentration was high. In summary, this study showed that NTA is a promising tool for measuring polydisperse samples of grape and wine macromolecules, and their aggregates under wine-like conditions. The implications for wine colloidal properties are discussed based on these results.

## 1. Introduction

Condensed tannins are among the most abundant macromolecules in red wine. They are extracted from grape skins and seeds during maceration and predominantly consist of condensed polymers of flavan-3-ols, at concentrations up to 4 mg/mL [1]. Under wine-like conditions, condensed tannins can aggregate and form colloidal dispersions, with hydrodynamic diameters in the magnitude of a few hundred to over a thousand nanometers [2,3]. The mean particle size of red wine colloids were shown to increase after 18 months of bottle aging, which was suspected to be responsible for the decrease in tannin concentration and molecular size observed in wines after aging [4]. These colloids may continue to aggregate and eventually precipitate and form sediment in wine bottles [5]. Thus, colloidal dispersion in wine is closely linked to wine mouthfeel perceptions and color stabilization, and warrants continuing research.

Polysaccharides in wine have been found to associate with condensed tannin non-covalently, through hydrogen bonding and hydrophobic interactions [6] and affect the colloid size evolution [7,8]. Polysaccharides are present in wine from 0.2 to 1.5 mg/mL, and consist predominately of neutral polysaccharides, which are mainly arabinogalactan-protein derived from the grape cell wall and mannoprotein derived from the yeast involved in fermentation [9]. Previous studies observed that polysaccharides mediate interactions between tannins and proteins [10,11,12], conferring impacts on wine mouthfeel [13], color stabilization [14] and fining (removal) of phenolic compounds [15]. For these reasons, polysaccharides have been used by the wine industry to improve wine composition and organoleptic characters. In Australia, two types of commercially manufactured polysaccharide additives are permitted in wine production: yeast mannoprotein and gum arabic (arabinogalactan) [16]. Supplementing wine with commercial polysaccharide products is likely to impact the colloidal stability of wine. It is therefore important to characterize the impact on colloidal dispersion in wine following the addition.

Characterizing interactions between grape- and wine-derived polysaccharides and tannins poses unique challenges, since both materials are very polydisperse. Grape skin tannins can be comprised of three to 83 flavan-3-ol subunits, while seed tannins are reported to have 2 to 16 subunits [1]. Wine polysaccharides are also heterogeneous, with molecular distribution reported to be between 5 and 800 kDa [9]. In addition, these macromolecules tend to interact and aggregate under wine conditions. Different fractions of macromolecules isolated from Pinot Noir wines, including tannins, polysaccharides and proteins, were shown to be highly polydisperse, with particle size distributions ranging from 20 to 500 nm [17]. Moreover, the properties of the dispersant have a significant impact on the macromolecular interaction, e.g., pH, ethanol concentration and ionic strength [8]. Thus, investigations into these interactions require non-invasive techniques, so as not to disrupt the non-covalent associations between particles, and at the same time, detect aggregate formation in a wine-like medium. Methods that have been employed to study polysaccharide and condensed tannin interactions include HPLC, nephelometry, Saturation-Transfer Difference-NMR, UV-Visible spectroscopy, dynamic light scattering, isothermal titration calorimetry, small-angle X-ray scattering and transmission electron microscopy [8,18,19,20,21,22].

Nanoparticle particle tracking analysis (NTA) is a relatively new technology (first commercialized in 2006) in nanoparticle characterization. It tracks the Brownian motion of individually recognized particles to deduce their hydrodynamic diameter. Since particles are individually analyzed, the size distribution in polydisperse samples is less skewed towards the large particles, hence giving more accurate results [23]. NTA has been applied to many food matrices and can handle non-aggressive solvents such as hydroalcoholic solutions [24]. Thus, NTA presents a promising technique to investigate the polydisperse colloidal dispersion in wine. In the current study, we aimed to investigate to what extent applying commercial polysaccharide additives would affect the colloidal state of wine, and by inference, the composition and organoleptic characters of wine. To this end, two polysaccharides purified from two commercial wine additives and a purified grape seed tannin fraction were combined in model wine solutions at two wine-like alcohol concentrations. To date, and to our knowledge, NTA has not been used to study tannin and polysaccharide interactions. Thus, the current study also aimed to evaluate the suitability of NTA for this type of investigation, corroborated by other techniques that have been successfully applied in this field.

## 2. Results and Discussion

### 2.1. Molecular Weight and Particle Size Distribution of Seed Tannin, Mannoprotein and Gum Arabic

Size exclusion chromatography showed that seed tannin (ST), mannoprotein (MP) and gum arabic (AG) had moderate polydispersity indices, from 1.8 to 2.3 (Table 1). However, the molecular weight ranges were substantially different. The molecular weight of ST ranged from 0.5 to 6 kg/mol, with a mean of 1.9 kg/mol, which approximated the degree of polymerization (DP) 6 [25]. In contrast, the two polysaccharides had much higher molecular weight ranges, 10–98 kg/mol and 48–322 kg/mol for MP and AG respectively, which were within the range that is typically observed for wine polysaccharides [9].

**Figure 1:**
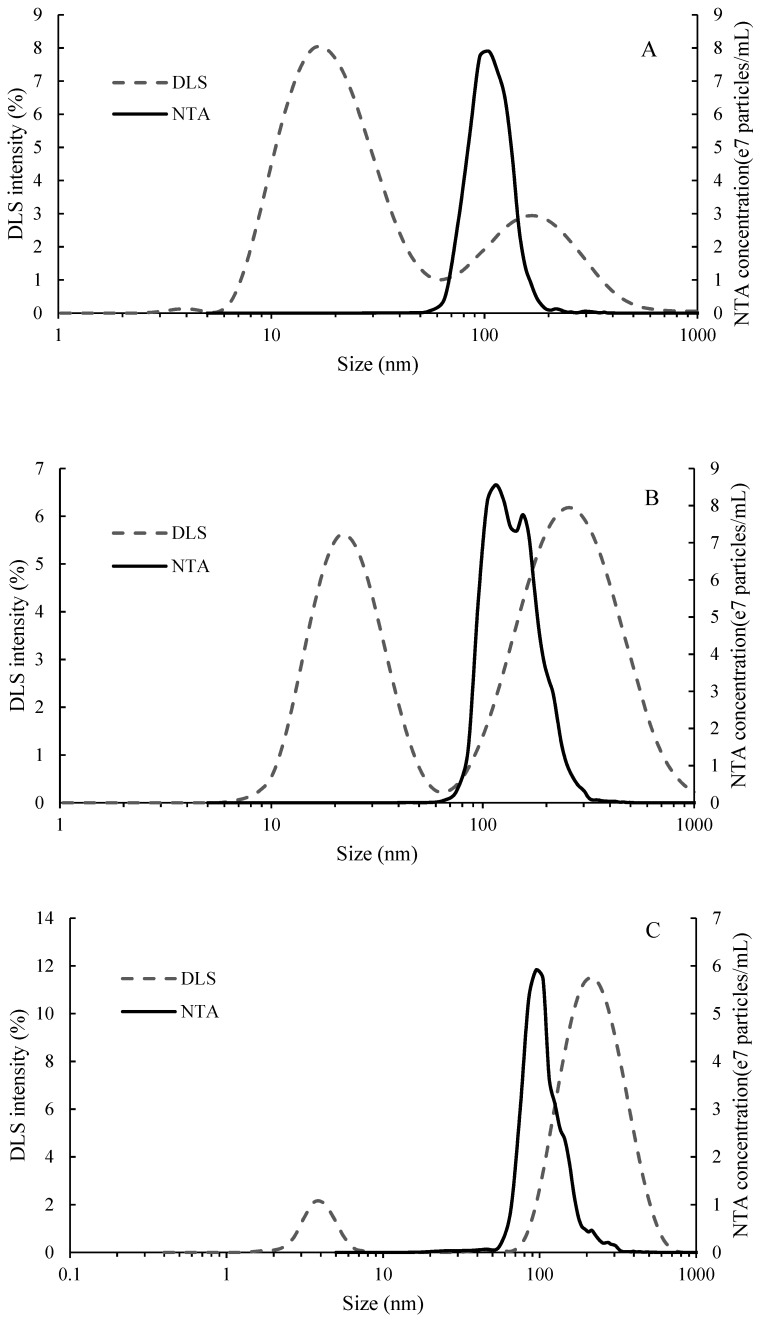
Particle size distribution of (**A**) MP, (**B**) AG and (**C**) ST, measured by dynamic light scattering (DLS) and nanoparticle tracking analysis (NTA).

The polydispersity index (PdI) of MP and AG determined by DLS were 0.5 and 0.9 respectively, while the cumulant fit error was higher than 0.005 for ST, indicating poor data quality or high sample polydispersity [26]. As such, the average particle size derived from cumulant analysis are not reported. Instead, intensity based particle size distributions, which are appropriate for polydisperse samples, are reported in Figure 1. DLS detected a peak between 10 and 60 nm for both MP and AG, as well as between 1 and 10 nm for ST, which were not detected by NTA. Both methods detected particles above 60 nm. This was possibly caused by the different detection limits of the two methods; for biological particles, the lower detection threshold for NTA is 60 nm ± 30% [27], while it is 1 nm for DLS [23]. The broad particle size distribution measured by DLS indicated that a range of aggregate sizes existed in the solutions. For MP and AG (Figure 1A,B), the intensity distribution showed that the particles of lower size were the primary component, considering intensity of light scattered by particles is proportional to the sixth power of its diameter. AGs isolated from commercial gum arabic generally have gyration radii between 20 and 30 nm [28], and tend to form particulate aggregates (≈100 nm) at concentrations as low as 0.1% in aqueous solution [29], where size increases with AG concentration. Commercial MP products also form aggregates when reconstituted in model wine solutions [30]. It was therefore considered that the observed aggregates >60 nm presented stable colloidal suspensions rather than insoluble material, which would be expected to have been removed during the centrifugation step [7].

The two groups of ST, at 4.1 and 256.6 nm (Figure 1C) could be detected by DLS, which were comparable to respective gyration radii of molecularly dissolved or aggregated grape seed tannins measured by small angle neutron scattering [31]. Using NTA, the major group of larger aggregates detected had a narrower distribution than determined by DLS, with a mean particle size of 91 nm. According to Zanchi and colleagues [31], 2% of native seed tannin loses solubility below 60% *v*/*v* ethanol, and represents a group of hydrophobic, oxidized tannins. Between 60% *v*/*v* ethanol and wine-like ethanol concentration (12% *v*/*v*) the bulk of tannin molecules are expected to remain in solution, while a sub-group (33%) forms metastable colloidal dispersions. It would be expected that the larger aggregates detected by DLS and NTA represented colloidally dispersed particles. According to Zanchi et al. [31,32], tannins which form aggregates between 12 and 60% *v*/*v* ethanol are preferentially solvated in ethanol, and that the proportion falling into this class may be enhanced by oxidation. The phloroglucinolysis conversion yield for ST was 67% (Supplementary Table S1), and it might be hypothesized that the uncharacterized portion indicates the presence of oxidized tannins in ST. This conversion yield is typical of grape seeds, which are thought to undergo oxidation in situ during ripening [25], but which may also potentially occur during the extraction process. In the literature, conversion yields of seed tannins were from 60 to 86% [33,34]. For comparison, a Tannat seed tannin of high conversion yield 86% [35] was compared with the ST used in this study, and produced the same particle distribution profile (data not shown). It would therefore be expected that any insoluble material would have been removed with the centrifugation step, as observed by Zanchi et al. [32] and that a portion of the oxidized tannin in the ST sample remained as a metastable colloidal dispersion in ethanol solution, while the remainder of the tannin was in solution (≤4 nm).

### 2.2. Interactions between Polysaccharides and Tannins Characterized by UV-Visible Spectrometry

**Figure 2:**
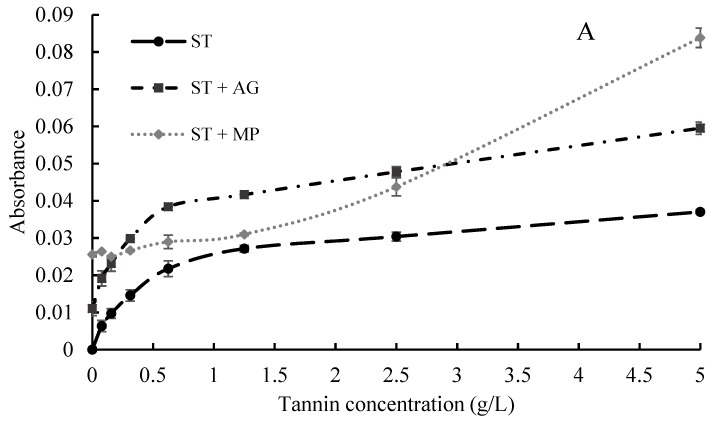

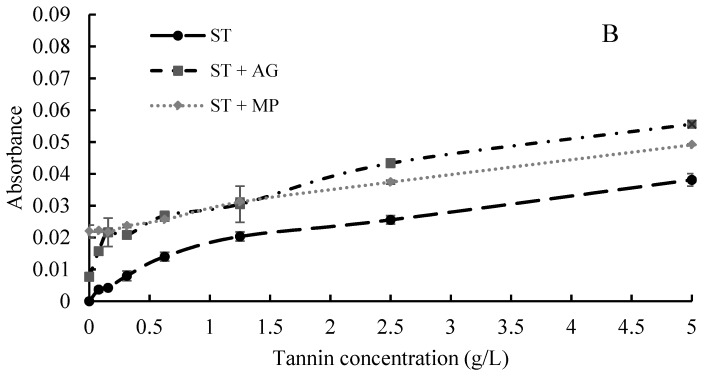
Absorbance (650 nm) of seed tannin from 0 to 5 mg/mL, with or without addition of polysaccharides in (**A**) 12% ethanol model wine and (**B**) 15% ethanol model wine.

Formation of aggregates between neutral polysaccharides and ST at a range of concentrations (0.065–5 mg/mL) were determined by measuring their absorbance at 650 nm. Since neither of these substances absorb light at this wavelength, the absorbance value is dominated by the light scattering intensity of particles, and therefore can serve as an indication of aggregate formation [6,20]. An initial absorbance in MP and AG solutions in the absence of ST indicated aggregates were naturally present in these solutions (Figure 2). For ST, a sharp increase in 650 nm absorbance was observed at lower ST concentrations, i.e., up to 1.25 mg/mL, followed by a steadier rise to 5 mg/mL, in both model wine solutions. This was likely due to decreased solubility at increasing concentrations. Absorbance of the mixture of ST and AG followed an identical trend to that of ST. In contrast to AG, the combination of MP and ST did not result in increases in absorbance at the lower ST concentrations. However, the 650 nm absorbance increased substantially in the MP and ST mixtures at the higher tannin concentrations of 2.5 and 5 g/mL in 12% model wine, indicating formation of highly scattering large particles (Figure 2A). Interestingly, in 15% model wine, the absorbance of the MP and ST combination increased evenly across the tannin concentration gradient. Strong increases in absorbance at 650 nm have been reported between a protein-rich arabinogalactan-protein (AGP) and procyanidins (DP 30) at high concentrations, although the absorbance reported was much higher than that found in the current study [6]. The UV-visible spectrometry measurement of MP, AG and ST has since been replicated in our lab (data not shown), and an analogous trend to the current study was found. The increase in absorbance at 650 nm for ST and MP in 12% model wine was further explored with NTA and DLS.

Absorbance at 280 nm was recorded in order to assess the impact of polysaccharide addition on phenolic content (retention or precipitation from solution). The absorbance values at 280 nm are reported in Supplementary Table S2. Regression analysis showed that the absorbance at 280 nm increased linearly (R^2^ > 0.99) with tannin concentrations, and was not affected by centrifugation or the alcohol concentration of the model wine. Although statistical analyses showed some differences in the absorbance at 280 nm between ST and the combination of ST and polysaccharides at certain tannin concentrations, there was a lack of consistency in the differences and no general trend could be attributed to the tannin concentration, polysaccharide type, centrifugation or ethanol concentration in the model wine (Supplementary Table S2). It was therefore assumed that the addition of polysaccharide did not influence the total phenolic concentration under the conditions used in the current study. No loss at 280 nm absorbance was observed in the ST and polysaccharide mixtures before or after mild centrifugation, indicating that centrifuging did not remove aggregates formed between tannin and polysaccharide. This was consistent with the report that aggregates formed between tannin and polysaccharide have low density and do not sediment with ultracentrifugation [19].

### 2.3. Binding Experiment Characterized by NTA

Based on the UV-vis spectroscopy results, two ST concentration points were further characterized by NTA and DLS: 1.25 and 5 mg/mL ST, combined with 0.5 mg/mL of either MP or AG, in both 12 and 15% model wine solutions.

**Figure 3:**
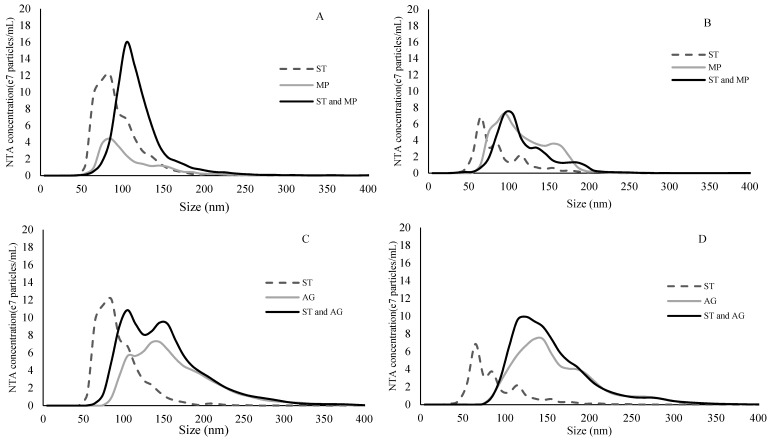
Size distribution of binding experiments between 1.25 mg/mL tannin and 0.5 mg/mL polysaccharides determined by nanoparticle tracking analysis. The curves were an average of 15 measurements. (**A**) ST and MP in 12% ethanol model wine; (**B**) ST and MP in 15% ethanol model wine; (**C**) ST and AG in 12% ethanol model wine; and (**D**) ST and AG in 15% ethanol model wine. All ST-containing solutions were diluted 1:10 with model wine prior to analysis.

**Figure 4:**
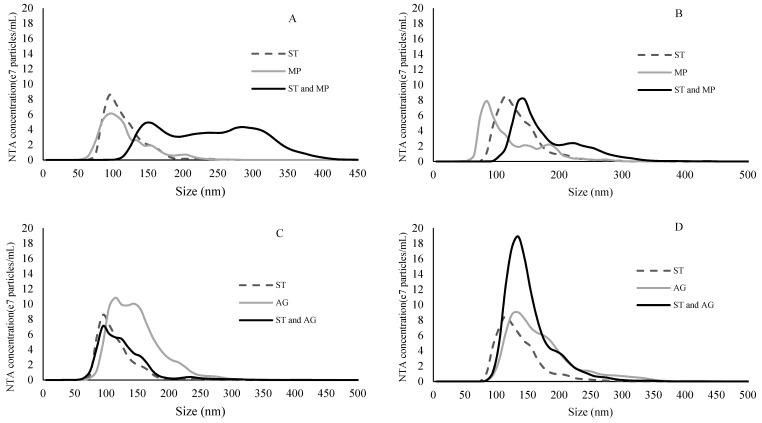
Size distribution of binding experiments between 5 mg/mL tannin and 0.5 mg/mL polysaccharides determined by nanoparticle tracking analysis. The curves are from an average of 15 measurements. (**A**) ST and MP in 12% ethanol model wine; (**B**) ST and MP in 15% ethanol model wine; (**C**) ST and AG in 12% ethanol model wine; and (**D**) ST and AG in 15% ethanol model wine. All ST-containing solutions were diluted 1:40 with model wine prior to analysis.

Number-weighted size distributions of particles of ST, MP, AG and their mixtures, were determined by NTA and compared (Figure 3 and Figure 4). Notably, the absolute concentrations (number of particles/mL) between samples were not compared in this instance because the camera settings and detection threshold were optimized for each sample and may have therefore affected particle recognition and count for each size class (and thus affect particle concentration). As a result, comparison of the distribution only aimed to identify shifts in particle sizes, in order to infer the formation of aggregates. NTA also determined particle size at the 10th, 50th and 90th percentiles of the distribution, as well as an overall mean. These numerical data were also reported for ease of comparison (Table 2).

At 1.25 mg/mL, ST particles were smaller than either of the polysaccharides, and when ST was combined with either polysaccharide type, the size distribution of the mixture shifted towards a higher average (Figure 3). The overall particle size of the ST and MP combination was slightly higher than that of MP alone in 12% model wine, but not in 15% model wine (Table 2). On the other hand, at both ethanol levels, the ST and AG combination or AG alone had almost identical size distributions, although AG alone had a slightly higher mean size than the mixture. At 5 mg/mL, ST formed larger particles than at 1.25 mg/mL, which were comparable or slightly larger than MP, but still smaller than AG (Table 2). The trend of particle size evolution between ST, AG and their mixtures observed at lower tannin concentrations generally held true in samples containing 5 mg/mL ST. However, obvious formation of aggregates between ST and MP could be detected at this tannin concentration. In particular, in 12% model wine, very large particles of between 250 and 400 nm could be found (Figure 4A). In general, the aggregate formation between AG and ST was relatively unaffected by either tannin or alcohol concentrations. In contrast, MP formed significantly larger aggregates at higher tannin concentrations which were further promoted as alcohol was lowered.

**Figure 5:**
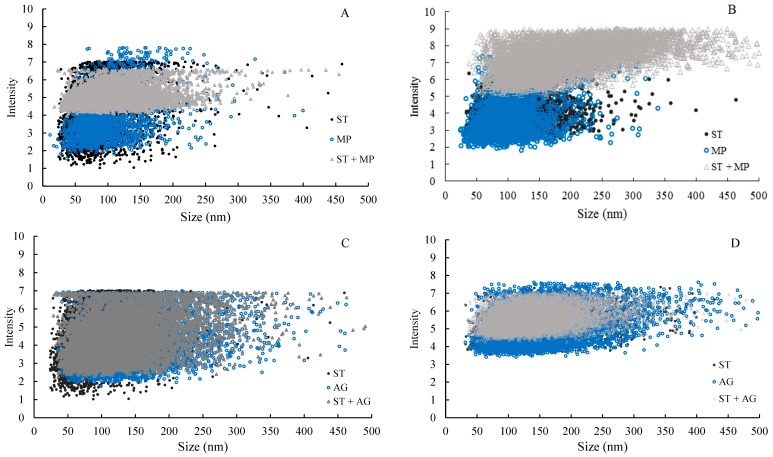
Size vs. light scattering intensity (arbitrary unit) for each tracked particle in nanoparticle tracking analysis. Only 1/5 of all tracked particles were included in the figures for clarity. (**A**) 1.25 mg/mL ST and 0.5 mg/mL MP in 12% ethanol model wine; (**B**) 5 mg/mL ST and 0.5 g/mL MP in 15% ethanol model wine; (**C**) 1.25 mg/mL ST and 0.5 mg/mL AG in 12% ethanol model wine; and (**D**) 5 mg/mL ST and 0.5 mg/mL AG in 15% ethanol model wine. ST solutions containing 1.25 mg/mL or 5 mg/mL respectively were diluted 1:10 or 1:40 with the corresponding model wine prior to analysis.

**Figure 6:**
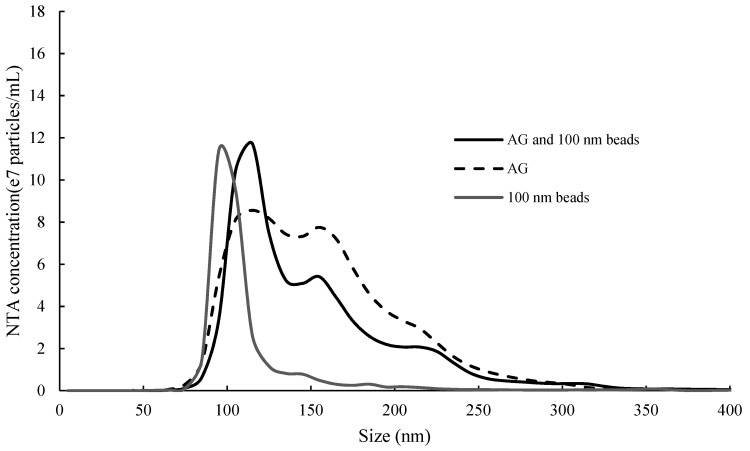
Size distribution of 100 nm polystyrene beads, AG and their mixture, determined by nanoparticle tracking analysis.

NTA also provided light-scattering intensity data for each tracked particle. Since camera settings affect the light scattering intensity recorded for samples, four sets of experiments where similar camera settings (Supplementary Table S3) were applied to all samples, were chosen for presentation in Figure 5. Some low-light scattering particles were detected in MP and ST samples individually, but only higher-light scattering particles were detected in the mixtures, especially when ST concentration was high (Figure 5A,B), indicating aggregation (interactions) between ST and MP. In contrast, no clear difference could be seen in the light scattering intensity of the mixture compared to AG and ST individually, with respect to ST and ethanol concentration (Figure 5C,D). It is interesting to note that, despite of increased particle light scattering intensity in the mixture of 1.25 mg/mL ST and MP in 12% ethanol model wine (Figure 5A), the particle size distribution shift was not apparent (Figure 3A and Table 2). Potentially, in this mixture, small oligomeric aggregates formed between the tannin and polysaccharide, but these were not bridged together to form larger aggregates due either to low tannin concentration or a low degree of polymerization [19,36]. However, further investigation was required to determine whether the disappearance of low-light scattering particles in ST and MP mixtures was truly due to tannin and polysaccharide aggregation or the limit of NTA measurement. It needed to be ascertained whether a mixture of two groups of particles of distinct yet similar sizes could be discriminated by NTA; i.e., if particles of larger size could potentially dominate the measurements. To further qualify this possibility, a small amount of 100 nm polystyrene beads was mixed with AG in 12% model wine solution and the size distribution was measured by NTA (Figure 6). In the size distribution profile of the mixture, both a distinctive peak of approximately 100 nm and a broader shoulder between 150 and 300 nm could be identified, representing the beads and the AG particles, respectively. Since the camera settings were similar between mixtures and their components (i.e., ST and polysaccharides), we concluded that if the smaller ST and MP particles were present in substantial quantities in the mixture, they should not have been entirely obscured by the larger species, and if present, should therefore have been detected.

The different behaviours between MP and AG towards ST was also explored with DLS. At both 0.125 and 0.5 mg/mL ST concentrations, the MP and ST combination resulted in a significantly higher light scattering intensity than was observed for the AG and ST combination (Supplementary Figure S1). In particular, at 0.5 mg/mL ST in 12% model wine solution, the light scattering intensity of the MP and ST combination was 7 times higher than that of the ST and AG combination. The DLS results confirmed those measured by NTA. Furthermore, DLS detected multiple particle size groups (peaks) and high PdI values in the AG and ST mixture (Supplementary Table S4), which were very similar in size to those observed in AG and ST separately (Figure 1). Conversely, the MP and ST combination showed only one apparent size group (PdI = 0.2), irrespective of ST and ethanol concentration. These results, together with results from NTA, strongly suggested that MP and ST formed aggregates under the current experimental conditions, while AG and ST had very weak interactions and if aggregates were formed, they were of low light scattering intensity with no apparent size evolution.

The weak interactions observed between AG and ST were in agreement with previous studies [3,6,8,10]. AG is composed of a ramified β(1 → 3)-d-galactose core that is highly branched at the 6 position with β(1 → 6) linked d-galactan side chains that are highly substituted with arabinose residues and to a lesser extent, glucuronic acid and rhamnose residues [29]. This highly branched structure and low mobility of galactan side chains may limit its ability to aggregate with tannin through hydrophobic interactions [6]. Application of commercial MP in red wine has been observed to either promote tannin aggregation and precipitation [37] or limit the loss of anthocyanin adducts [14]. Similarly, in model wine solution, a commercial MP (10% protein *w*/*w*, molecular weight distribution 14–500 kDa) has been observed to form large aggregates with grape and wine tannins [7], consistent with the current results. In contrast, MP purified from wine, in particular the low molecular weight fractions (1.6–3.5%, protein *w*/*w* with narrow molecular weight distribution around 51 to 62 kDa), limited seed tannin aggregation through steric hindrance, resulting in a smaller overall particle size [3,8]. The MP used in the current study had a molecular weight distribution between 10 and 98 kDa (Table 1) with a protein content at 11% of the dry weight. It appeared that the different behaviours towards ST were more related to protein content than molecular size. It has been shown that mannoproteins have significantly lower affinity to tannin than yeast-derived protein and bovine serum albumin [7,38]. Furthermore, between two wine AGP fractions, only the one with slightly higher protein content (3.6 vs. 0.8%) could form aggregates with procyanidins of DP 30 [6]. If such a small proportion of protein could induce a substantial difference in aggregate formation between polysaccharides and tannins, it might also explain the different behaviours between MP and AG in the current study, since MP had a higher protein content than AG (10 vs. 1.4%). This would potentially have significant implications for wine production. This is because native wine polysaccharide composition is highly variable and capable of impacting on tannin composition and subsequently wine astringency [13,39]. Furthermore commercial polysaccharide supplements could also be added to wine, as discussed previously, which adds further unknowns to the system. It has been shown that the protein content of commercial MP products can range from 10 to 50% [40]. Therefore, the choice of product, with varying concentration of active ingredients and other by-products, could have a great impact on the final wine colloidal state, potentially affecting the colour and organoleptic characters of wine post-addition.

Polysaccharide is thought to be important in mediating tannin and protein aggregation, through one or more mechanisms: (i) polysaccharides form ternary complexes with tannin-protein aggregates and thereby increase their solubility; and (ii) polysaccharides bind with tannin and thus limit access of protein [18,41]. The current study showed that for certain polysaccharides, the second mechanism is in effect. In the future, different types of protein could be introduced into this system to explore the competition between polysaccharides and various proteins for tannin binding in greater detail.

Lower ethanol concentration was found to promote aggregate formation between ST and MP. Ethanol preferentially solvates tannin and reducing ethanol content in an aqueous solution decreases tannin solubility [2,31]. Moreover, lowering ethanol concentration favors tannin-tannin or tannin-protein aggregation through hydrophobic interactions [31,42]. The effect of ethanol on tannin and mannoprotein interactions have been reported [8,38]. However, none of these studies reported an effect when the concentration differences between treatments were as small as those used in the current study (3%). Ethanol concentrations between 12 and 15% are typically found in red table wine. From a sensory point of view, a 4% increase in alcohol concentration could reduce astringency and enhance bitterness (two mouthfeel characters highly associated with wine polyphenolic composition) in model wine solutions [43,44]. Thus, the effect of ethanol on the colloidal state of wine macromolecules and its implication for wine sensory characters warrants further investigation.

## 3. Materials and Methods

### 3.1. Preparation of Polysaccharide and Tannin Materials

Cabernet Sauvignon grapes were harvested at the pre-veraison stage (green berries, pea size) from a commercial vineyard in South Australia, and frozen at −80 °C until used. Frozen berries were partially defrosted while kept on ice, and the seeds removed using a scalpel. A sample of 100 g of seeds was extracted overnight in 200 mL of 70% *v*/*v* aqueous acetone containing 10 mg/mL ascorbic acid. Extracts were filtered through a 0.5 mm mesh to remove solids and the recovered solution was centrifuged at 1730× *g*. Acetone was removed from the supernatant under vacuum at 35 °C and the remaining aqueous solution was lyophilized. The dried extract was reconstituted in 50 mL of 60% *v*/*v* HPLC grade aqueous methanol containing 0.05% *v*/*v* trifluoroacetic acid (TFA) and then applied (∼18.3 mL/min) to a glass column (Michel-Miller, 300 × 21 mm, Vineland, NJ, USA) containing Sephadex LH20 chromatography resin (Amersham, Uppsala, Sweden) to an approximate bed volume of 93 mL, previously equilibrated with the loading solvent. The monomeric phenolics, organic acids and sugars were removed by application of 300 mL of 60% *v*/*v* aqueous methanol containing 0.05% *v*/*v* TFA. ST was recovered following application of 250 mL of 70% *v*/*v* aqueous acetone containing 0.05% *v*/*v* TFA. The eluted ST fraction was concentrated under reduced pressure at 35 °C to remove organic solvents and then lyophilized to a dry powder. ST was stored under nitrogen at −20 °C until used. The subunit composition of ST was determined by HPLC following acid catalysis in the presence of excess phloroglucinol [45,46]. The molar proportion of each subunit, mean degree of polymerization and mass conversion are reported in Supplementary Table S1.

Two polysaccharides were prepared from commercial supplements used in vinification. The MP product was a highly pure cell wall extract from *Saccharomyces cerevisiae* (Mannofeel, Laffort Australia, Adelaide, Australia) while the arabinogalactan was purified from a commercial blend of gum arabic and grape tannin (Surli Vitis, Enartis Pacific, Melbourne, Australia), by removing the associated phenolic compounds with three extractions in 70% acetone (monitored by HPLC with a UV-vis detector at 280 nm). Both polysaccharides were dialyzed against 4 changes of MilliQ water using a 7 kDa cut-off membrane (SnakeSkin dialysis tubing, Thermo Scientific, Rockford, IL, USA), and then lyophilized. The subunit composition of polysaccharide was determined according to a published method [17]. Briefly, 1 mg/mL polysaccharide solution was hydrolysed in 2 M TFA for 3 h at 100 °C. Hydrolysates were dried in vacuo and reconstituted in 0.4 mL of Milli-Q water and mixed 1:1 with an aqueous internal standard solution comprising 0.6 mM ribose and deoxy-glucose (Sigma Aldrich, St. Louis, MO, USA). Mixtures were derivatized with 1-phenyl-3-methyl-5-pyrazolone (PMP) and analysed by RP-HPLC, using a C18 column (Kinetex, 2.6 µm, 100 Ǻ, 100 × 3 mm). The HPLC instrumentation and mobile phase gradient were as reported previously (Bindon, et al. 2016). Total nitrogen content was measured by the analytical services unit of the Commonwealth Scientific and Industrial Research Organization (CSIRO, Adelaide, Australia), using a TruMAC (Leco Corporation, Saint Joseph, MI, USA); powdered polysaccharides were combusted in an atmosphere of oxygen and nitrogen determined as gaseous N2 by thermal conductivity detection. The composition of the products are reported in Supplementary Table S5.

Two model wine solutions (4 mg/mL tartaric acid, pH adjusted to 3.4 with analytical grade sodium hydroxide) containing ethanol levels at 12 and 15% (*v*/*v*) were used in the current study. The ionic strength was estimated at 20 mM based on the relative abundance of tartaric acid and its ionized forms at pH 3.4. Solutions were filtered through a 0.2 µm membrane (Durapore, Merck Millipore, Cork, Ireland) before use. For all experiments, ST, MP and AG were reconstituted in model wine solution at gravimetric concentrations (*w*/*v*).

### 3.2. Macromolecule Characterization

#### 3.2.1. Size Exclusion Chromatography Analysis

The molecular weight distribution of ST, MP and AG were determined by size exclusion chromatography (SEC). ST was analysed with an HPLC (Agilent 1100, Agilent Technologies Australia Pty. Ltd., Melbourne, Australia), using the gel permeation chromatography (GPC) method originally reported by Kennedy and Taylor [46], with modifications described by Bindon and Kennedy [47]. The retention times at 10% and 90% ST elution by volume were compared against the standard curve to derive lower and upper ranges for molecular weight, while the retention time at 50% elution was used to determine mean molecular weight. In addition, the polydispersity index (PdI) was calculated by dividing weight average molecular weight (M_r_) by number average molecular weight (M_n_).

The size distribution of polysaccharides was analysed using an Agilent 1260 HPLC system fitted with a Yarra SEC-4000 column connected to a Yarra SEC-2000 column (silica resin, 3 µm, 300 × 7.8 mm, Phenomenex, Torrance, CA, USA). The mobile phase was 0.1 M NaNO3 with a flow rate of 1.2 mL/min for a 22.5 min run time, at 40 °C. Refractive index signals were analysed with ChemStation GPC data analysis software Rev B.01.01 (Agilent Technologies Australia Pty. Ltd., Melbourne, Australia). Polysaccharide molecular weight was determined by comparing samples to a calibration curve developed with a series of pullulan standards of known molecular weight (Shodex, Showa Denko K.k, Japan): P800 (708 kDa), P400 (344 kDa), P200 (200 kDa), P100 (107 kDa), P50 (47.1 kDa), P20 (21.1 kDa), P10 (7.6 kDa) and P5 (5.9 kDa). Each standard was run 5 times to check for retention time shift, which was not observed (data not shown). A third order polynomial curve was established between elution volume and molecular weight, with an R^2^ of 0.9973 (Supplementary Figure S2). The mean and range of molecular weight, as well as PdI of polysaccharides, were determined in the same way as described for ST.

#### 3.2.2. DLS Analysis

A Malvern Zetasizer Nano ZS (Malvern Instruments Ltd, Worcestershire, UK), equipped with a 4 mW He-Ne laser at a wavelength of 633 nm was used in the current study. Instrument control and data analysis were performed with Zetasizer software (version 7.10). For each measurement, the temperature was maintained at 25 °C, and the angle of detection was set at 90°. Measurement position, attenuator level and measurement duration were all set to be automatically optimized by the software.

Particle size (hydrodynamic diameter) was determined using the Stokes-Einstein equation:

(1) $$d\left(H\right)=\;\frac{kT}{3\pi \eta D}$$

where *k* is Bolzmann’s constant; *T* is absolute temperature; *η* is dispersant viscosity and *D* is diffusion coefficient. Viscosity was determined with Zetasizer software based on the molar content of ethanol in solutions and were 1.367 cP and 1.518 cP respectively for 12 and 15% ethanol model wines. *D* was determined by fitting an autocorrelation function to the exponential with two different algorithms: (i) cumulants analysis, which determined the mean particle size (Z-ave) and polydispersity index (PdI); and (ii) non-negative least squares (NNSL) analysis, which generated intensity weighted size distribution, using the ‘general purpose mode’ in this instance. Disposable low volume cuvettes with a pathlength of 10 mm were used for measurements.

#### 3.2.3. NTA Analysis

Nanoparticle tracking analysis (NTA) was performed on a Nanosight NS300 (Malvern Instruments Ltd, Worcestershire, UK), equipped with a 635 nm laser and a scientific CMOS camera. NTA 3.0 software was used for instrument control and data analysis. The data was collected in the form of 60-second videos captured by the camera. The sample chamber was maintained at 25 °C and a syringe pump was used to keep a continuous flow of sample through the flow cell at 7 µL/min for the duration of measurement.

For each individual sample, settings (screen gain, camera level and focus) were manually adjusted to optimize visualization of the particles and thereafter kept identical for all video repetitions of the same sample (Supplementary Table S3). Detection threshold, which determined the minimal brightness of pixels to be considered for tracking, was also adjusted post-acquisition to minimize noise as well as to maintain a particle per frame count appropriate for analysis (10–100 particles per frame). Settings were kept consistent for all video repetitions of the same sample. The NTA software measured the mean square displacement from the centre of the particle’s scatter as it moved from frame to frame in the collected videos. The hydrodynamic diameter of particles were calculated from the modified Einstein-Stokes equation:

(2) $$\bar{{\left(x,\;y\right)}^{2}}=\;\frac{4kTt}{3\pi d\eta }$$

where $\bar{{\left(x,y\right)}^{2}}$ is the mean square of displacement; *k* is Bolzmann’s constant; *T* is absolute temperature; *t* is time; *d* is the hydrodynamic diameter and *η* is dispersant viscosity [27]. The viscosity values were identical to those used in DLS measurements.

#### 3.2.4. System Qualification for NTA and DLS Instruments

NIST-traceable polystyrene latex bead standards (100, 200 and 400 nm) were supplied by Malvern Instruments Ltd (Worcestershire, UK). The standards were dispersed in 0.01 M KCl. For DLS measurements, all three bead standards were diluted 1:10. For NTA measurements the dilution factors were according to the instrument supplier’s manual, i.e., 1:1000 dilution for 100 nm, 1:100 dilution for 200 nm and 1:10 dilution for 400 nm. All samples were measured 5 times, by either DLS or NTA. For each system, the accuracy of measurements of 100 nm and 200 nm beads were within those specified by the International Standardization Organization [27,48] and were in good agreement with one another (Supplementary Table S6). Although the measurements for 400 nm beads deviated more from the stated size, accuracy was still within 10% for each method.

#### 3.2.5. Particle Size of Tannin and Polysaccharide Determined by DLS and NTA

For particle size distribution determined by either DLS or NTA, ST, MP and AG were reconstituted by vortexing in model wine containing 12% ethanol. All solutions were centrifuged at 3273× *g* for 5 min before measurements. Polysaccharides and ST were reconstituted in model wine at 0.5 mg/mL and 0.125 mg/mL respectively, for NTA characterization. At these concentrations no excessive scattering was observed while all particles could be clearly visualized under the scientific CMOS camera. Fifteen video repetitions were taken for each sample. DLS analysis required samples to be much more concentrated. Higher concentrations were trialled on DLS to find a working concentration that was closest to those used for NTA analysis. It was found that 4 mg/mL was the minimal concentration at which sufficient scattered light could be detected by the DLS instrument during a measurement, i.e., a mean count rate higher than 20 kilo counts per second and therefore this concentration was chosen. Each sample was measured five times.

### 3.3. Characterization of Interactions between Polysaccharides and Tannins

#### 3.3.1. UV-visible Spectroscopy Analysis

The aggregation between polysaccharides and tannins at various concentrations were measured as absorbance at 650 nm by UV-visible spectrometry. This assay was adapted and modified from a previous study [6]. ST was reconstituted in the two model wine solutions at 10 mg/mL, while MP and AG were reconstituted separately at 1 mg/mL. The control samples consisted of 1 mL of diluted ST solution of 0, 0.078, 0.156, 0.313, 0.625, 1.25, 2.5 and 5 mg/mL (*w*/*v*), along the columns on a 96-well plate (1.1 mL volume, Axygen, Adelab, Adelaide, Australia). For the treatment samples, 0.5 mg/mL of either MP or AG was added to the ST solutions, while maintaining the same tannin concentrations and volumes as control samples. Both control and treatment samples were prepared in duplicate. The plates were sealed with a compatible silicone sealing mat, vigorously shaken and stored at 22 °C for 24 h. Thereafter, 200 µL from each well was then transferred into a clear 96-well cycloolefine plate (Greiner, Sigma-Aldrich, Sydney, Australia) and the absorbance at 650 nm recorded with a SpectraMax M2 Microplate reader (Molecular Devices, Melbourne, Australia). In addition, a 20 µL sample aliquot was diluted with 980 µL of 1 M HCl solution and 280 nm absorbance was recorded to determine total phenolics, according to Mercurio, Dambergs, Herderich, and Smith [49]. The plate was then centrifuged at 3273× *g* for 5 min, and another 20 µL sample diluted with 980 µL of 1 M HCl and the absorbance measured at 280 nm.

#### 3.3.2. NTA and DLS Analyses

ST was reconstituted at 2.5 mg/mL or 10 mg/mL, while AG and MP were both reconstituted at 1 mg/mL, in each model wine solution. The ST solution was mixed in equal parts (750 µL each) with each polysaccharide solution and all stock solutions were diluted 1:1 with model wine to create two series of samples with final concentrations of (i) 1.25 mg/mL tannin, 0.5 mg/mL polysaccharide and their mixtures and (ii) 5 mg/mL tannin, 0.5 mg/mL polysaccharide and their mixtures. The solutions were sealed in 1.5 mL Eppendorf tubes and kept at 22 °C for 24 h before being centrifuged at 16,100× *g* for 5 min to remove insoluble particles, if any, as shown by others [7,32]. The samples were then used directly for DLS analysis. However, for NTA, the supernatants containing ST, individually or combined with either polysaccharide type, were diluted 1 in 10 with model wine solutions for the low concentration series and 1 in 40 for the high concentration series, while supernatants containing only polysaccharide were measured without dilution. The samples for NTA and DLS measurements were individually prepared. For all samples, 15 video repetitions were recorded by NTA and four replicates were performed by DLS.

#### 3.3.3. Tannin Solubility

It was considered that large tannin aggregates might be present due to insolubility, according to previous studies [31,32]. The Cabernet Sauvignon seed tannin isolate used for the experiments (mass conversion 67% *w*/*w* by phloroglucinolysis) was prepared in 15% acidified ethanol (model wine) as described above, or made up directly in pure ethanol, then diluted to 15% *v*/*v* with water containing tartaric acid, equivalent to the model wine matrix used for experimentation. NTA of the samples was then compared when analysed neat, or centrifuged at 16,100 g for 10 min which according to Zanchi and colleagues [32] would be adequate to sediment insoluble, unstable tannin particles. For tannin solutions directly reconstituted in model wine, no difference in particle concentration was observed following centrifugation, but there was a small increase in particle size. For tannin reconstituted in ethanol and then diluted, higher particle concentrations were observed than for direct reconstitution which were lost (50%) upon centrifugation (data not shown), indicating this mode of reconstitution produced colloidal instability. Hence, samples were reconstituted directly in model wine for all experiments.

## 4. Conclusions

This study presents the first application of NTA for the characterization of tannin and polysaccharide interactions in wine-like media. NTA was able to assess shifts in size distribution in aggregated colloids, following addition of commercial polysaccharide supplements into model wine solutions containing grape seed tannins. The light scattering intensity of individually tracked particles can provide additional insight into aggregate formation. The NTA results were confirmed by DLS and UV-vis analysis. The two polysaccharides, MP and AG, derived from commercial winemaking additives used in wine production, were considerably different in colloidal behaviour when mixed with ST. MP formed larger, highly light scattering aggregates, while AG had only weak interactions with ST, with no clear indications of aggregate formation. A 3% ethanol reduction was found to increase aggregate size for MP, but had no impact on AG.

## Figures and Tables

**Table 1:** Mean and range of molecular weight (M_r_) and polydispersity index (PdI) determined by size exclusion chromatography.

Material	Mean M_r_ (kg/mol)	PdI	M_r_10 (kg/mol)	M_r_90 (kg/mol)
Mannoprotein (MP)	33.6 ± 1.9	2.25 ± 0.07	9.6 ± 0.3	97.9 ± 8.8
Gum arabic (AG)	132.5 ± 1.0	1.75 ± 0.01	47.6 ± 0.4	321.6 ± 1.4
Seed tannin (ST)	1.92 ± 0.02	2.14 ± 0.01	0.5 ± 0.01	6.0 ± 0.09

All values are means of 3 measurements ± standard error. M_r_10 and M_r_90 stand for molecular weight at the 10th and 90th percentiles of elution respectively.

**Table 2:** Particle size of seed tannin (ST) combined with commercial mannoprotein (MP) or gum arabic (AG) at 0.5 mg/mL measured by nanoparticle tracking analysis.

ST ^a^ (mg/mL)	Model Wine ^b^	Treatment	Percentile and Mean Particle Size by NTA (nm)				Treatment	Percentile and Mean Particle Size by NTA (nm)			
			D10	D50	D90	Mean		D10	D50	D90	Mean
	12%	ST	56.4 ± 0.8	79.1 ± 1.3	121.9 ± 2.5	91.4 ± 1.4	ST	56.4 ± 0.8	79.1 ± 1.3	121.9 ± 2.5	91.4 ± 1.4
		MP	62.8 ± 0.6	87.7 ± 0.9	158.3 ± 5.0	105.7 ± 1.0	AG	96.0 ± 0.9	143.5 ± 0.8	226.9 ± 2.9	160.1 ± 1.0
1.25 ^c^		ST + MP	80.2 ± 0.8	104.0 ± 0.9	152.5 ± 3.3	116.5 ± 1.0	ST + AG	85.8 ± 1.3	133.1 ± 1.2	214.1 ± 2.8	147.6 ± 1.4
	15%	ST	48.3 ± 0.9	71.4 ± 1.9	124.0 ± 4.9	85.7 ± 1.8	ST	48.3 ± 0.9	71.4 ± 1.9	124.0 ± 4.9	85.7 ± 1.8
		MP	68.7 ± 1.0	101.1 ± 1.2	155.6 ± 1.3	113.2 ± 0.8	AG	98.2 ± 1.4	141.0 ± 2.1	223.1 ± 4.3	157.5 ± 1.7
		ST + MP	73.3 ± 0.9	99.0 ± 0.8	160.1 ± 3.3	113.9 ± 1.0	ST + AG	97.3 ± 0.9	134.5 ± 1.0	209.9 ± 3.4	151.0 ± 0.9
	12%	ST	78.3 ± 1.2	99.6 ± 1.2	147.3 ± 4.7	112.5 ± 1.3	ST	78.3 ± 1.2	99.6 ± 1.2	147.3 ± 4.7	112.5 ± 1.3
		MP	71.5 ± 1.3	100.8 ± 2.1	160.9 ± 4.9	113.4 ± 2.0	AG	93.8 ± 0.7	132.6 ± 1.8	193.4 ± 4.1	144.2 ± 1.9
5 ^d^		ST + MP	134.5 ± 2.8	229.2 ± 3.5	316.2 ± 3.8	231.2 ± 3.1	ST + AG	77.3 ± 1.0	108.6 ± 1.5	160.9 ± 6.0	121.4 ± 2.0
	15%	ST	90.5 ± 1.3	119.7± 1.7	170.4 ± 2.9	131.4 ± 1.5	ST	90.5 ± 1.3	119.7± 1.7	170.4 ± 2.9	131.4 ± 1.5
		MP	68.0 ± 1.0	100.2 ± 4.2	184.1 ± 7.7	120.7 ± 3.2	AG	107.2 ± 1.1	148.2 ± 2.2	237.9 ± 5.6	167.1 ± 2.2
		ST + MP	117.9 ± 1.5	153.3 ± 2.5	249.5 ± 6.2	176.1 ± 2.5	ST + AG	105.1 ± 1.0	132.9 ± 1.0	192.8 ± 2.6	147.3 ± 1.3

Values are means of 15 measurements ± standard error. ^a^ Seed tannin concentration in the solutions. ^b^ Ethanol concentrations in the model wine solutions (*v*/*v*). ^c^ All solutions containing seed tannin were diluted 1:10 with model wine prior to analysis. ^d^ All solutions containing seed tannin were diluted 1:40 with model wine prior to analysis.

## Supplementary Materials

The following are available online at https://www.mdpi.com/1420-3049/24/11/2100/s1. Table S1. Subunit composition of ST; Table S2. Absorbance (280 nm) of ST at different concentrations, either individually or combined with 0.5 mg/mL polysaccharide (MP or AG), in 12% and 15% ethanol model wine, before and after centrifugation; Table S3. Camera shutter and gain settings for binding experiment characterized by NTA; Table S4. Polydispersity index (PdI) and intensity weighted mean particle size distribution determined by dynamic light scattering. The samples contained ST at either 1.25 or 5 mg/mL, combined with 0.5 mg/mL of either MP or AG; Table S5. Monosaccharide residue composition of polysaccharide following hydrolysis; Table S6. Mean size and size distribution of polystyrene beads determined by dynamic light scattering and nanoparticle tracking analysis; Figure S1. Comparison of light scattering intensity (measured as derived count of photons) of ST combined with either MP or AGP, in both 12% and 15% model wine solutions; Figure S2. Calibration curve for polysaccharide molecular weight based on size exclusion chromatography.
