# Supplementary material for: Applying Nanoparticle Tracking Analysis to Characterize the Polydispersity of Aggregates Resulting from Tannin–Polysaccharide Interactions in Wine-Like Media
Source: Molecules. 2019 Jun 3;24(11):2100. doi: 10.3390/molecules24112100 (PMC6600421; doi:10.3390/molecules24112100)
Supplement: Supplementary file 1 [file molecules-24-02100-s001.pdf]

Supplementary Data

Applying Nanoparticle Tracking Analysis to Characterize Interactions between Tannin and Polysaccharide in Wine-like Media

Sijing Li[1,2], Kerry L. Wilkinson[1,2], Agnieszka Mierczynska-Vasilev[3], and Keren A. Bindon[3*],

[1] The University of Adelaide, School of Agriculture, Food and Wine, PMB 1, Glen Osmond, SA 5064, Australia

[2] The Australian Research Council Training Centre for Innovative Wine Production.

[3] The Australian Wine Research Institute, PO Box 197, Glen Osmond, SA 5064, Australia

***Corresponding author**: Dr. Keren Bindon, telephone: +61 8 83136640, facsimile: +61 8 83136601, email: keren.bindon@awri.com.au

Table S1. Subunit composition of ST.

Tannin	MC[b] (%)	mDP[c]	Extension subunits[a]				Terminal subunits[a]		
			EGC-P	C-P	E-P	ECG-P	C	E	ECG
ST	67.3	5.18	0.0	7.5	58.9	14.3	7.4	3.0	8.9

[a] Molar fractions (%) of procyanidin subunits with the following abbreviations: (-P) , phloroglucinol adduct of extension subunit; EGC, (–)-epigallocatechin; C, (+)-catechin; EC, (–)-epicatechin; ECG, (–)-epicatechin-3-*O*-gallate.

[b] Mass conversion derived from percent recovery of procyanidin subunits by phloroglucinolysis based on the gravimetric mass.

[c] Mean degree of polymerisation in epicatechin units.

Table S2. Absorbance (280 nm) of ST at different concentrations, either individually or combined with 0.5 mg/mL polysaccharide (MP or AG), in 12% and 15% ethanol model wine, before and after centrifugation.

Treatment	Tannin concentration (mg/mL)						
	0.078	0.156	0.313	0.625	1.25	2.5	5
12% model wine solution, before centrifugation.							
ST + MP	0.017 a	0.031	0.092 a	0.177	0.366	0.750 b	1.514
ST + AG	0.023 a	0.031	0.088 b	0.161	0.341	0.858 a	1.604
ST	0.019 b	0.028	0.085 c	0.174	0.340	0.660 c	1.348
P-value	0.020	0.197	0.005	0.104	0.596	0.013	0.072
Significant	Yes	No	Yes	No	No	Yes	No
12% model wine solution, after centrifugation							
ST + MP	0.011	0.026	0.074	0.164	0.354	0.694	1.396
ST + AG	0.011	0.026	0.079	0.164	0.345	0.814	1.498
ST	0.013	0.027	0.083	0.176	0.359	0.732	1.420
P-value	0.109	0.327	0.380	0.172	0.435	0.202	0.751
Significant	No	No	No	No	No	No	No
15% model wine solution, before centrifugation							
ST + MP	0.013	0.029	0.081	0.166	0.355	0.688	1.382
ST + AG	0.013	0.028	0.083	0.170	0.350	0.806	1.486
ST	0.013	0.028	0.085	0.178	0.360	0.731	1.408
P-value	0.868	0.362	0.760	0.344	0.689	0.208	0.775
Significant	No	No	No	No	No	No	No
15% model wine solution, after centrifugation							
ST + MP	0.015	0.029	0.082	0.162	0.342	0.697 b	1.307
ST + AG	0.021	0.030	0.081	0.173	0.364	0.802 a	1.386
ST	0.018	0.027	0.081	0.168	0.353	0.729 b	1.344
P-value	0.052	0.071	0.651	0.072	0.162	0.015	0.342
Significant	No	No	No	No	No	Yes	No

Values are means of duplicates. Values followed by different letters within column are significantly different ($p \leq 0.05$, one way ANOVA followed by Fisher's LSD).

Table S3. Camera shutter and gain settings for binding experiment characterized by NTA.

12% model wine			15% model wine		
	camera shutter	camera gain		camera shutter	camera gain
ST 1.25	800	350	ST 1.25	1000	400
MP 0.5	600	250	MP 0.5	600	350
AG 0.5	800	350	AG 0.5	800	350
MP ST 1.25	800	350	MP ST 1.25	800	350
AG ST 1.25	800	350	AG ST 1.25	800	350
ST 5	800	350	ST 5	600	350
MP 0.5	600	350	MP 0.5	800	350
AG 0.5	600	300	AG 0.5	600	250
MP ST 5	250	250	MP ST 5	450	250
AG ST 5	600	350	AG ST 5	600	350

The highlighted samples are presented in Figure 6.

Table S4. Polydispersity index (PdI) and intensity weighted mean particle size distribution determined by dynamic light scattering. The samples contained ST at either 1.25 or 5 mg/mL, combined with 0.5 mg/mL of either MP or AG.

Treatment	PdI	Peak 1[a] (nm)	Peak 2[a] (nm)	Peak 3[a] (nm)
12% model wine solution				
ST5[b] + MP	0.20 ± 0.01	193 (100)	-	-
ST5 + AG	0.99 ± 0.01	265 (53)	29 (40)	4.7 (7)
ST1.25[c] + MP	0.20 ± 0.00	86 (100)	-	-
ST1.25 + AG	0.69 ± 0.07	242 (53)	27 (46)	-
15 % model wine solution				
ST5 + MP	0.25 ± 0.01	131 (100)	-	-
ST5 + AG	1.00 ± 0.00	212 (56)	25 (37)	3.5 (6)
ST1.25 + MP	0.23 ± 0.00	74 (100)	-	-
ST1.25 + AG	0.65 ± 0.08	221 (58)	26 (42)	-

[a]Results are reported as mean particle size of the each peak detected by dynamic light scattering. Values in parentheses are percentage of total scattered light represented by each peak. [b] Solutions containing 1.25 mg/mL ST were diluted 1:10 with the corresponding model wine prior to analysis. [c]Solutions containing 5 mg/mL ST were diluted 1:40 with the corresponding model wine prior to analysis

Table S5. Monosaccharide residue composition of polysaccharide following hydrolysis.

Polysaccharide type	PRT[a] (%)	PL MC[b] (%)	Monosaccharide composition[c]						
			Man	Rha	GlcA	GalA	Glu	Gal	Ara
MP	11.3	74.5	85.8	n.d.	n.d.	n.d.	14.2	n.d.	n.d.
AG	1.4	77.7	1.2	3.7	7.4	1.5	0.8	36.4	49.1

[a] Protein as percentage of the gravimetric mass, estimated by multiplying total nitrogen by a factor of 6.25.

[b] Mass conversion derived from the percent recovery of monosaccharide residue based on gravimetric mass.

[c] Molar fraction (%) of monosaccharide residues following hydrolysis, with the following abbreviations: Man, mannose; Rha, rhamnose; GalA, galacturonic acid; GlcA, glucuronic; Gal, galactose; Ara, arabinose.

Table S6. Mean size and size distribution of polystyrene beads determined by dynamic light scattering and nanoparticle tracking analysis.

	DLS		NTA	
Bead size (nm)	Z-ave (nm)	PdI	mean (nm)	SD (nm)
100 nm	100.9 ± 0.3	0.03 ± 0.01	101.2 ± 1.5	13.6 ± 2.9
200 nm	202.8 ± 3.3	0.02 ± 0.01	189.8 ± 0.5	17.0 ± 3.4
400 nm	433.0 ± 3.6	0.05 ± 0.02	371.7 ± 1.3	28.1 ± 3.5

Values are means of duplicates. Values followed by different letters within column are significantly different ($p \leq 0.05$, one way ANOVA followed by Fisher's LSD).

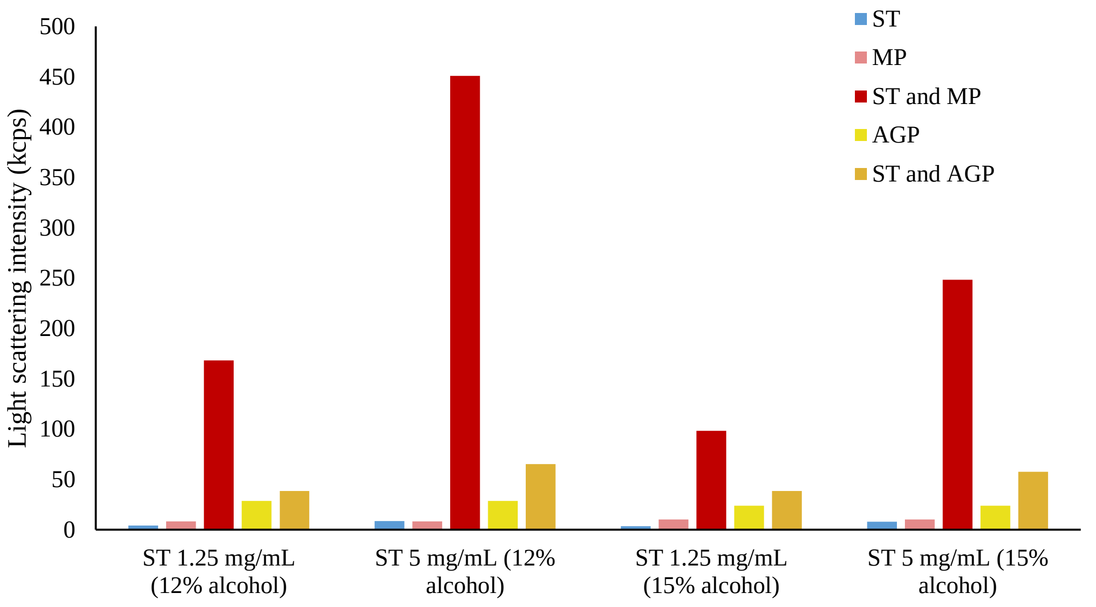

Figure S1. Comparison of light scattering intensity (measured as derived count of photons) of ST combined with either MP or AGP, in both 12% and 15% model wine solutions. Solutions containing 1.25 mg/mL or 5 mg/mL ST were diluted 1:10 or 1:40 respectively with the corresponding model wine prior to analysis.

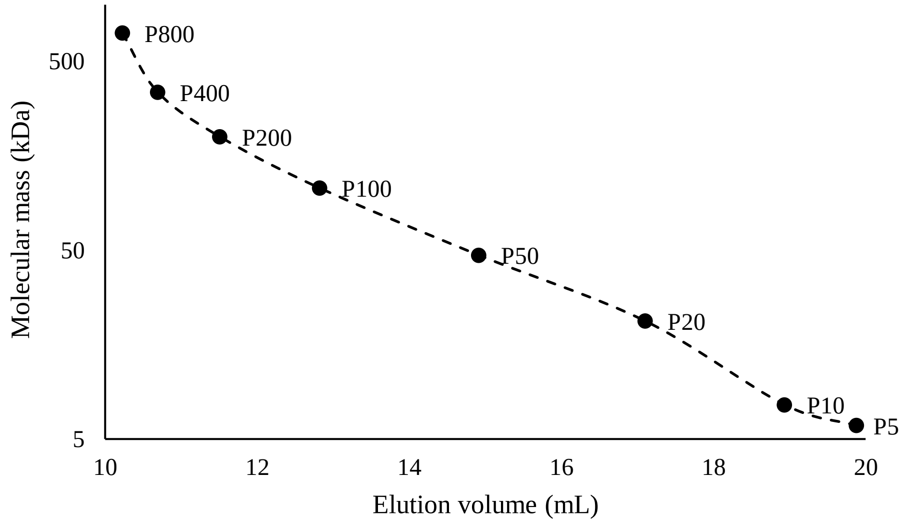

Figure S2. Calibration curve for polysaccharide molecular weight based on size exclusion chromatography.
